# Interleukin‐1α‐Mediated Activation of TNF‐α/NF‐κB Signaling Confers Resistance to Osimertinib in *EGFR* ‐Mutant Non‐Small‐Cell Lung Cancer

**DOI:** 10.1111/1759-7714.70360

**Published:** 2026-07-17

**Authors:** Wira Winardi, Yoichiro Mitsuishi, Hironari Matsuda, Fumiyuki Takahashi, Daisuke Hayakawa, Koichiro Kanamori, Ikuko Nakamura, Aditya Wirawan, Moulid Hidayat, Adityo Wibowo, Takehito Shukuya, Ken Tajima, Takuo Hayashi, Shinji Kohsaka, Kazuhisa Takahashi

**Affiliations:** ^1^ Department of Respiratory Medicine Juntendo University Faculty of Medicine and Graduate School of Medicine Tokyo Japan; ^2^ Department of Human Pathology, Graduate School of Medicine Juntendo University Tokyo Japan; ^3^ Division of Cellular Signaling National Cancer Center Research Institute Tokyo Japan

**Keywords:** cycling cancer persister cells, drug resistance, *EGFR*‐mutant non‐small‐cell lung cancer, osimertinib

## Abstract

**Background:**

Lung cancer is the leading cause of cancer‐related deaths worldwide. Although osimertinib is more effective than previous‐generation drugs, its efficacy is often limited by both primary and acquired resistance. Cycling cancer persister cells (CPCs) have recently emerged as a transient, drug‐tolerant subpopulation that may contribute to early resistance to targeted therapies. However, their biological characteristics and role in osimertinib resistance remain poorly understood.

**Methods:**

We established osimertinib‐resistant CPCs derived from epidermal growth factor receptor (*EGFR)*‐mutant non‐small‐cell lung cancer (NSCLC) cell lines PC9 and HCC827. RNA sequencing (RNA‐seq) was performed to identify CPC‐specific transcriptional programs.

**Results:**

RNA‐seq analyses demonstrated that the tumor necrosis factor alpha (TNF‐α)/nuclear factor‐κB (NF‐κB) signaling pathway was upregulated in osimertinib‐resistant PC9 cells (PC9‐CPCs), with interleukin‐1 alpha (IL‐1α) being the most significantly overexpressed gene. Treatment with IL‐1α increased TNF‐α and NF‐κB p65 expression in PC9 parental cells, whereas IL‐1α inhibition decreased TNF‐α expression in PC9‐CPCs. Consequently, the inhibition of either IL‐1α or TNF‐α restored the sensitivity of *EGFR*‐mutant NSCLC cells to osimertinib. Immunohistochemical analysis confirmed that NF‐κB expression was higher in tumor specimens from patients who had developed osimertinib resistance than in specimens collected before treatment.

**Conclusion:**

CPCs exhibit a distinct inflammatory phenotype driven by IL‐1α–driven activation of the TNF‐α/NF‐κB axis, representing an alternative mechanism underlying osimertinib resistance. Targeting IL‐1α or TNF‐α may provide a promising strategy to overcome resistance in patients with *EGFR*‐mutant NSCLC.

## Graphical abstract

Cycling persister cells drive osimertinib resistance in *EGFR*‐mutant lung cancer through an autocrine IL‐1α–TNF‐α–NF‐κB circuit; blocking this axis restored drug sensitivity in vitro and in vivo.

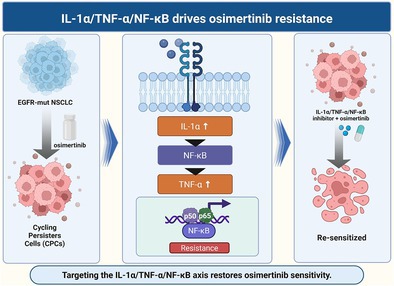

## 1 Introduction

Non‐small‐cell lung cancer (NSCLC) is the most prevalent type of lung cancer, and adenocarcinoma is the most common histological subtype, affecting both smokers and non‐smokers alike [1]. Mutations in the epidermal growth factor receptor (EGFR) tyrosine kinase are typical in NSCLC adenocarcinoma cases, especially in Asian populations [2]. Advanced NSCLCs that contain characteristic mutations in EGFR, exon 19 deletions or exon 21 L858R mutations, demonstrate high sensitivity to EGFR tyrosine kinase inhibitors (TKIs). Osimertinib is a third‐generation irreversible EGFR‐TKI with greater potency against both canonical *EGFR*‐activating mutations and the T790M‐resistance mutation. Initial studies demonstrated that osimertinib exhibited a superior objective response rate and progression‐free survival (PFS) compared to standard chemotherapy in patients with T790M‐driven acquired resistance to first‐generation and second‐generation EGFR‐TKIs [3].

Subsequently, osimertinib was compared with gefitinib or erlotinib as first‐line therapy for patients with advanced *EGFR*‐mutant NSCLC in the FLAURA trial, and improved median PFS and prolonged overall survival (OS) were demonstrated [4, 5]. Despite the efficacy of highly potent third‐generation EGFR‐TKIs, acquired resistance to osimertinib treatment is inevitable. A number of resistance mechanisms related to the first‐line treatment with osimertinib have been reported, including both EGFR‐dependent and EGFR‐independent mechanisms [6, 7]. However, those underlying early resistance to osimertinib as a first‐line therapy remain to be elucidated. In this study, we established osimertinib‐resistant *EGFR*‐mutant NSCLC cells and investigated the non‐genetic mechanisms underlying resistance to osimertinib, focusing on “cycling cancer persister cells (CPCs),” also known as “drug‐tolerant expanding persisters.” They form a rare subset of cancer persisters that can re‐enter the cell cycle under constitutive drug treatment [8, 9]. RNA‐sequencing results indicated a pivotal role of interleukin‐1α‐mediated activation of tumor necrosis factor alpha (TNF‐α)/nuclear factor‐κB (NF‐κB) signaling in the development of osimertinib resistance. Therefore, the TNF‐α/NF‐κB signaling pathway possibly represents a viable target for therapeutic intervention, with the potential to avert disease recurrence.

## 2 Methods

### 2.1 Cell Culture and Reagents

NSCLC cell lines, PC9 and HCC827, were used in this study. PC9 cells were kindly provided by Dr. Kazuto Nishio (Department of Genome Biology, School of Medicine, Kindai University, Osaka) and were established at the Tokyo Medical University (Tokyo, Japan), as described previously [10]. HCC827 cells were obtained from the American Type Culture Collection (Manassas, VA, USA). The cells were cultured at 37°C in Roswell Park Memorial Institute (RPMI) 1640 medium supplemented with 10% fetal calf serum. Osimertinib was purchased from AstraZeneca (Cambridge, UK).

### 2.2 Transcriptome Sequencing

Total RNA was extracted from fresh‐frozen samples using RNA‐Bee (Tel‐Test Inc., Gainesville, FL, USA), treated with DNase I (Thermo Fisher Scientific, Waltham, MA, USA), and subjected to poly(A)‐RNA selection prior to cDNA synthesis. The library used for RNA‐seq was prepared using a NEBNext Ultra Directional RNA Library Prep Kit (NEB, Ipswich, MA, USA), in accordance with the manufacturer's protocol. Sequencing was conducted from both ends of each cluster using a HiSeq 2500 or NextSeq platform (Illumina, San Diego, CA, USA). The RNA‐seq data were aligned to the hg19 reference genome using the TopHat software (v2.0.9; https://ccb.jhu.edu/software/tophat/index.shtml). The expression level of each gene was calculated using Cufflinks (v2.1.1; https://cole‐trapnell‐lab.github.io/cufflinks/).

### 2.3 RNA Expression Analysis

Differential gene expression analysis was performed using the edgeR pipeline via the Galaxy platform [11, 12]. Genes were excluded from further analysis if the samples had normalized counts less than 0.5 count per million in at least two samples. Differentially expressed genes were determined by establishing cutoff points using a log fold change (log FC) > 1 and a *p* < 0.05. The heatmap was generated using the HeatMap Image in GenePattern (http://www.broad.mit.edu/genepattern) [13]. Kaplan–Meier survival curves were generated using KM plotter [14]. For lung adenocarcinoma, *IL1A* was analyzed using the 210118_s_at probe and TNFA using the 207113_s_at probe. Patients were dichotomized into “high” and “low” expression groups based on the median expression value, and differences in overall survival were assessed using the log‐rank test. The analysis was performed using data accessed on May 31, 2026.

### 2.4 Gene Set Variation Analysis (GSVA)

We employed GSVA to calculate IL‐1α and TNF signaling scores [15]. The R software package was used to compute GSVA scores based on the transcripts per million (TPM) value. The gene sets representing IL‐1α and TNF activity were selected from the Molecular Signatures Database (MSigDB) [16].

### 2.5 Chemosensitivity Assay

Briefly, cells were planted in 96‐well plates in quadruplicate at a density of 1 × 10^3^ cells/well. The cells were cultivated for 72 h in the absence or presence of osimertinib at varying concentrations. Cell viability was assessed using a Cell Counting Kit‐8 (CCK‐8; FUJIFILM Wako Pure Chemical Corporation, Osaka, Japan) according to the manufacturer's instructions.

### 2.6 Cell Viability Assay

PC9 or HCC827 cells were seeded in a 96‐well plate (1 × 10^3^ cells/100 μL media/well) and incubated overnight before being treated with the appropriate drugs at 100 ng/mL for 72 h under regular cell culture conditions. Live‐cell cultures were treated with CCK cell viability reagent for 3 h at 37°C after incubation. Subsequently, cell viability was assessed according to the protocol established by the manufacturer of the CCK‐8 reagent.

### 2.7 Western Blotting

Nuclear protein was extracted using NE‐PER nuclear and cytoplasmic extraction reagent (Thermo Fisher Scientific, Waltham, MA, USA) according to the manufacturer's protocol, with the addition of protease and phosphatase inhibitors (Roche, Basel, Switzerland). The detergent‐compatible protein assay was used to determine the protein concentration (Bio‐Rad, Hercules, CA, USA). The proteins were loaded onto Mini‐PROTEAN TGX Precast Gels (Bio‐Rad, Hercules, CA, USA). After blocking with Can Get Signal (Toyobo Life Science, Osaka, Japan) blocking reagent, the blots were incubated overnight with the following primary antibodies: anti‐NF‐κB p65 antibody (1:1000; Cat. no. D14E12; Cell Signaling Technology, Danvers, MA, USA) and anti‐histone H3 antibody (1:1000; Cat. no. ab8580; Abcam, Cambridge, UK). The appropriate horseradish peroxidase‐conjugated secondary antibody was used to incubate the membranes (GE Healthcare, Little Chalfont, Buckinghamshire, UK), and enhanced chemiluminescence was used to detect the results. Each dilution was prepared using CanGet Signal Immunoreaction Enhancer Solution (Toyobo Life Science, Osaka, Japan). The western blot signals were captured using the ChemiDoc and ChemiDoc MP imaging systems, in conjunction with the Image Lab Touch Software, developed by Bio‐Rad (Hercules, CA, USA).

### 2.8 Quantitative Real‐Time Polymerase Chain Reaction (qPCR)

The RNeasy Mini Kit (Qiagen, Hilden, Germany) was used to extract total RNA from the cells. The Revertra cDNA synthesis kit (Toyobo Life Science, Osaka, Japan) was used to generate cDNA from 1 mg of RNA according to the manufacturer's protocol. The Fast SYBR Green Master Mix (Applied Biosystems, Foster City, CA, USA) was used for qPCR under the following thermal cycling conditions: denaturation at 95°C for 20 s followed by 40 amplification cycles (denaturation at 95°C for 3 s, annealing and extension at 60°C for 30 s), with melt‐curve analysis. Glyceraldehyde‐3‐phosphate dehydrogenase (GAPDH) was used as an internal control.

The primer sequences used for the analyses were as follows:

**IL1A**

*Forward*: 5′‐AGATGCCTGAGATACCCAAAACC‐3′

*Reverse*: 5′‐CCAAGCACACCCAGTAGTCT‐3′

**TNF**

*Forward*: 5′‐CCTCTCTCTAATCAGCCCTCTG‐3′

*Forward*: 5′‐GAGGACCTGGGAGTAGATGAG‐3′

**GAPDH**

*Forward*: 5′‐CATGTTCGTCATGGGGTGAACCA‐3′

*Reverse*: 5′‐AGTGATGGCATGGACTGTGGTCAT‐3′

### 2.9 Enzyme‐Linked Immunosorbent Assay (ELISA)

Cells were cultivated in serum‐free medium for 72 h. After incubation, the supernatant was collected. IL‐1α levels were determined using commercially available Quantikine ELISA kits (R&D Systems, Madrid, Spain) according to the manufacturer's instructions.

### 2.10 CRISPR‐Cas9‐Mediated Knockout (KO)

For the CRISPR/Cas9‐mediated knockout, two short guide RNAs (sgRNAs) were selected from the human sgRNA library. Single guide oligonucleotides used in this study were as follows:

sgScramble (F): 5′‐CACCGGGCCCGCATAGGATATCGC‐3′, sgScramble (R): 5′‐AAACGCGATATCCTATGCGGGCCC‐3′, sgIL1A #1 (F): 5′‐CACCGATGGTGGTAGTAGCAACCAA‐3′, sgIL1A #1 (R): 5′‐AAACTTGGTTGCTACTACCACCATC‐3′, sgIL1A #2 (F): 5′‐CACCGAGCCGTGAGGTACTGATCAT‐3′, sgIL1A #2 (R): 5′‐AAACATGATCAGTACCTCACGGCTC‐3′, sgTNFR1 #1 (F): 5′‐CACCGACCAGTCCAATAACCCCTG‐3′, sgTNFR1 #1 (R): 5′‐AAACCAGGGGTTATTGGACTGGTCC‐3′, sgTNFR1 #2 (F): 5′‐CACCAAGACCAAAGAAAATGACCA‐3′, sgTNFR1 #2 (R): 5′‐AAACTGGTCATTTTCTTTGGTCTTC‐3′.

Oligonucleotide pairs were annealed and cloned into a lentiCRISPR v2 lentiviral vector (Addgene plasmid #52961) [17]. For lentiviral production, Lenti‐X 293 T cells were transfected with lentiCRISPR v2, psPAX2 (Addgene #12260), and pMD2.G (Addgene plasmid #12259) at a 1:1:10 ratio using 2× HEPES‐buffered saline (2× HBS), then cultured in Dulbecco's modified Eagle medium supplemented with 10% FCS and 50 units/mL penicillin/streptomycin. The medium was replaced 24 h after transfection to remove precipitates, and viral supernatants were collected at 48 h and filtered through a 0.45‐μm filter. The filtered viral medium was added to PC9‐CPCs, followed by spinfection at 32°C–35°C and 2300 rpm for 2 h. Cells were then cultured under standard conditions and selected with 2 μg/mL puromycin until all non‐transduced control cells were eliminated. During the CRISPR/Cas9 knockout procedure, both control (sgScramble) and knockout CPCs were transiently maintained in osimertinib‐free RPMI medium during lentiviral transduction and puromycin selection (approximately 10–14 days) prior to functional assays. This common exposure history applies equally to both groups, preserving the validity of the relative comparison between control and knockout cells.

### 2.11 Establishment of Osimertinib‐Resistant Cells

This study generated the resistant cell model following an established procedure using PC9 and HCC827 cell lines. Both PC9 and HCC827 are lung adenocarcinoma cell lines that have a deletion in exon 19 of the *EGFR* gene and are sensitive to EGFR‐TKI, including osimertinib. The cells were exposed to high concentrations of osimertinib (1 μM) for 3 months to establish osimertinib‐resistant cells. They were cultivated in RPMI medium supplemented with 10% FCS and antibiotics. The osimertinib‐containing medium was replaced every 3 days. A chemosensitivity assay was performed to evaluate the half maximal inhibitory concentration (IC_50_) at the end of the procedure.

### 2.12 Immunohistochemical Analysis

Paraffin‐fixed tissue samples were sliced into 3‐μm‐thick sections, deparaffinized in xylene, and then rehydrated in an ethanol solution. Subsequently, they were autoclaved at 120°C for 10 min to retrieve the antigen. Endogenous peroxidase was blocked with 0.3% H_2_O_2_ for 15 min. Following an overnight incubation at 4°C with rabbit anti‐NF‐κB p65 antibody (1:400, catalog no. D14E12, Cell Signaling Technology), the sections were washed, incubated with HRP‐linked secondary antibodies at room temperature for 45 min, and developed in liquid 3,3′‐diaminobenzidine before counterstaining with hematoxylin.

### 2.13 Xenograft Experiment

The experimental subjects were male athymic nu/nu mice, aged 4–6 weeks, procured from Sankyo Labo Service Corporation (Tokyo, Japan). The mice were housed in a pathogen‐free barrier room in Juntendo University's Animal Care Facility and handled according to aseptic standards. All procedures were approved by the Animal Committee at Juntendo University. Prior to the commencement of the experimental phase, the mice were allowed at least 7 days to acclimate to their new environment. During this acclimatization phase, the mice had unrestricted access to both food and water.

A total of 1 × 10^6^ PC9‐CPCs were suspended in extracellular matrix gel derived from Engelbreth‐Holm‐Swarm murine sarcoma (Sigma‐Aldrich) and injected subcutaneously into the right flank of nude mice. Each mouse received an injection of 0.2 mL containing 1 × 10^6^ cells using a 26‐gauge needle. Drug administration was initiated when the average tumor volume reached approximately 0.015 cm^3^.

The mice were divided into four groups and treated with either vehicle, osimertinib alone, BAY11‐7082 alone, or a combination of osimertinib and BAY11‐7082. Osimertinib was administered orally by gavage at a dose of 25 mg/kg/day, 5 days per week. BAY11‐7082 was administered at a dose of 15 mg/kg by direct intratumoral injection every alternate day, starting from a tumor volume of approximately 150 mm^3^.

The mice were monitored daily, and tumor size was measured thrice per week using digital calipers for a period of 14 days. Tumor volume was calculated using the oblong ellipsoid formula: volume = (width^2^ × length)/2, where length is the longest dimension and width is the dimension perpendicular to the length.

### 2.14 Statistical Analysis

GraphPad Prism was used for statistical analysis (GraphPad Software, La Jolla, CA). The two‐tailed Student's *t*‐test and analysis of variance were used to compare values. Data are presented as mean ± standard deviation. Statistical significance is indicated as follows: *p < 0.05, **p < 0.01, ***p < 0.001, and ****p < 0.0001.

## 3 Results

### 3.1 Establishment of Osimertinib‐Resistant PC9 and HCC827 Cell Lines

**FIGURE 1:**
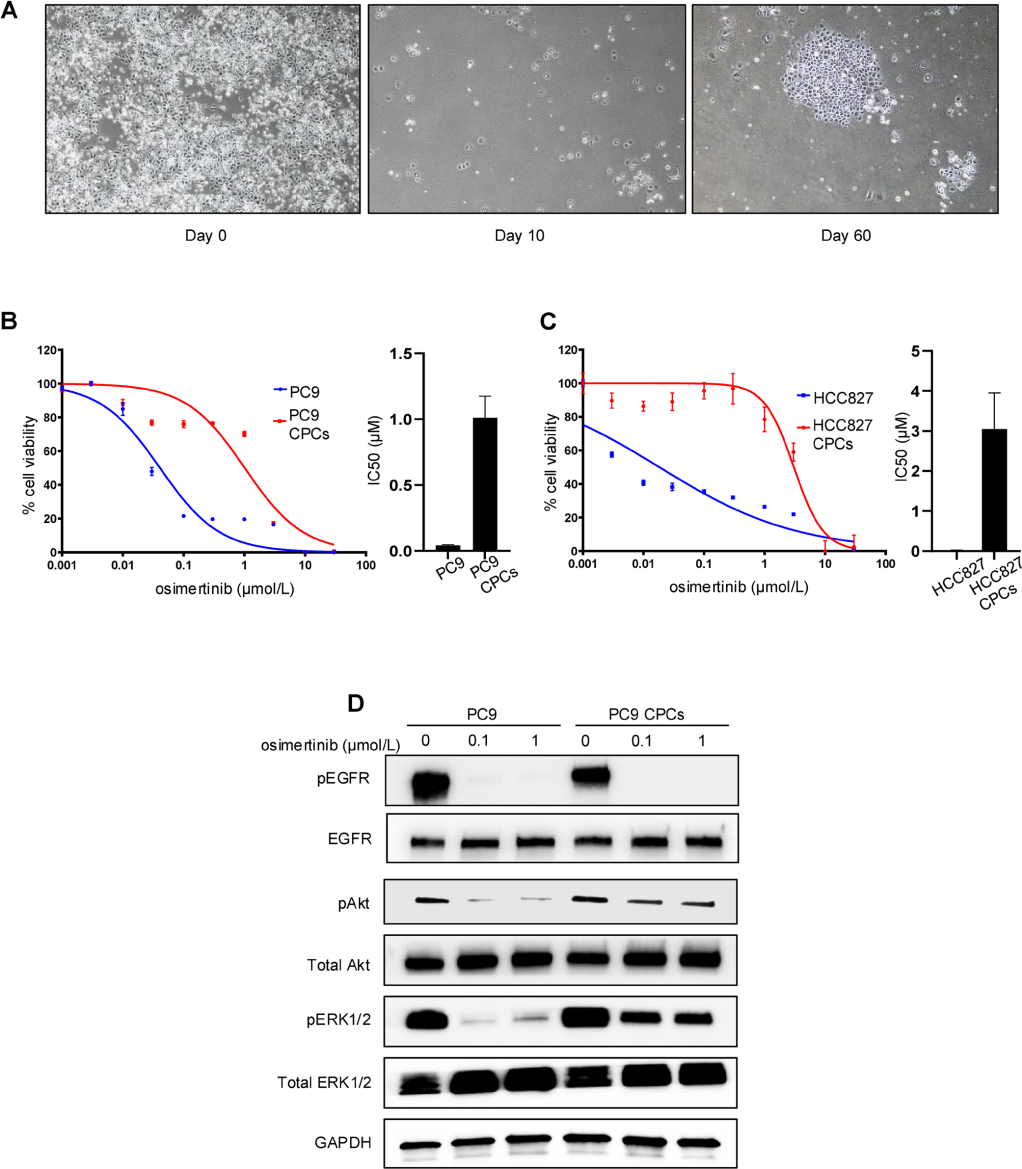
Generation of osimertinib‐resistant CPCs from PC9 and HCC827 cell lines. (A) PC9 cells were treated with 1 μM osimertinib for 90 days. After 10 days of treatment, most cells died, and a small population remained quiescent. After 2 months, the cells started to regrow despite treatment with a high concentration of osimertinib. (B and C) Survival curves of PC9/HCC827 cells and PC9/HCC827‐CPCs treated with indicated osimertinib concentrations for 72 h. The bar graph corresponds to 50% inhibition concentration (IC_50_). (D) Western blot profiling of EGFR–Akt–ERK pathway activity in PC9 parental and PC9‐CPCs cells treated with increasing doses of osimertinib. Data are presented as mean ± SD and are representative of three independent experiments. CPCs, cycling cancer persister cells; EGFR–Akt–ERK, epidermal growth factor receptor–protein kinase B–extracellular signal‐regulated kinase; SD, standard deviation.

To determine the mechanism of osimertinib resistance in primary treatment, we continuously exposed cancer cells to high concentrations (1 μM) of osimertinib for 3 months to establish resistant cells. After 10 days of treatment, most cells died while some remained and stayed quiescent. This subpopulation was referred to as drug‐tolerant persisters (DTPs). After 2 months of osimertinib treatment, the cells continued to proliferate despite receiving high doses of the drug. These are referred to as drug‐tolerant extended persisters and have been recently identified as CPCs that form a small fraction of the initial DTP population (Figure 1A). The chemosensitivity assay at the end of the experiment revealed that the IC_50_ value of osimertinib‐resistant PC9‐CPCs was 24 times greater than that of the parental PC9 cells (Figure 1B). A comparable resistance phenotype was observed in HCC827‐CPCs, which also exhibited markedly elevated IC_50_ values compared with parental HCC827 cells (Figure 1C). In addition, western blot results showed that phosphorylation levels of both protein kinase B (AKT) and extracellular signal‐regulated kinase (ERK) were markedly higher in PC9 CPCs than in parental cells upon treatment with osimertinib. The results demonstrated that osimertinib treatment did not effectively inhibit the AKT and ERK signaling pathways in PC9 CPCs, thereby contributing to its limited efficacy (Figure 1D). Whole‐exome sequencing of PC9 and HCC827 CPCs did not detect any known resistance‐associated mutation, including EGFR C797S, MET amplification, or alterations in KRAS, PIK3CA, or BRAF (data not shown).

### 3.2 TNF Signaling via NF‐κB Gene Signature Was Linked to Osimertinib Resistance

**FIGURE 2:**
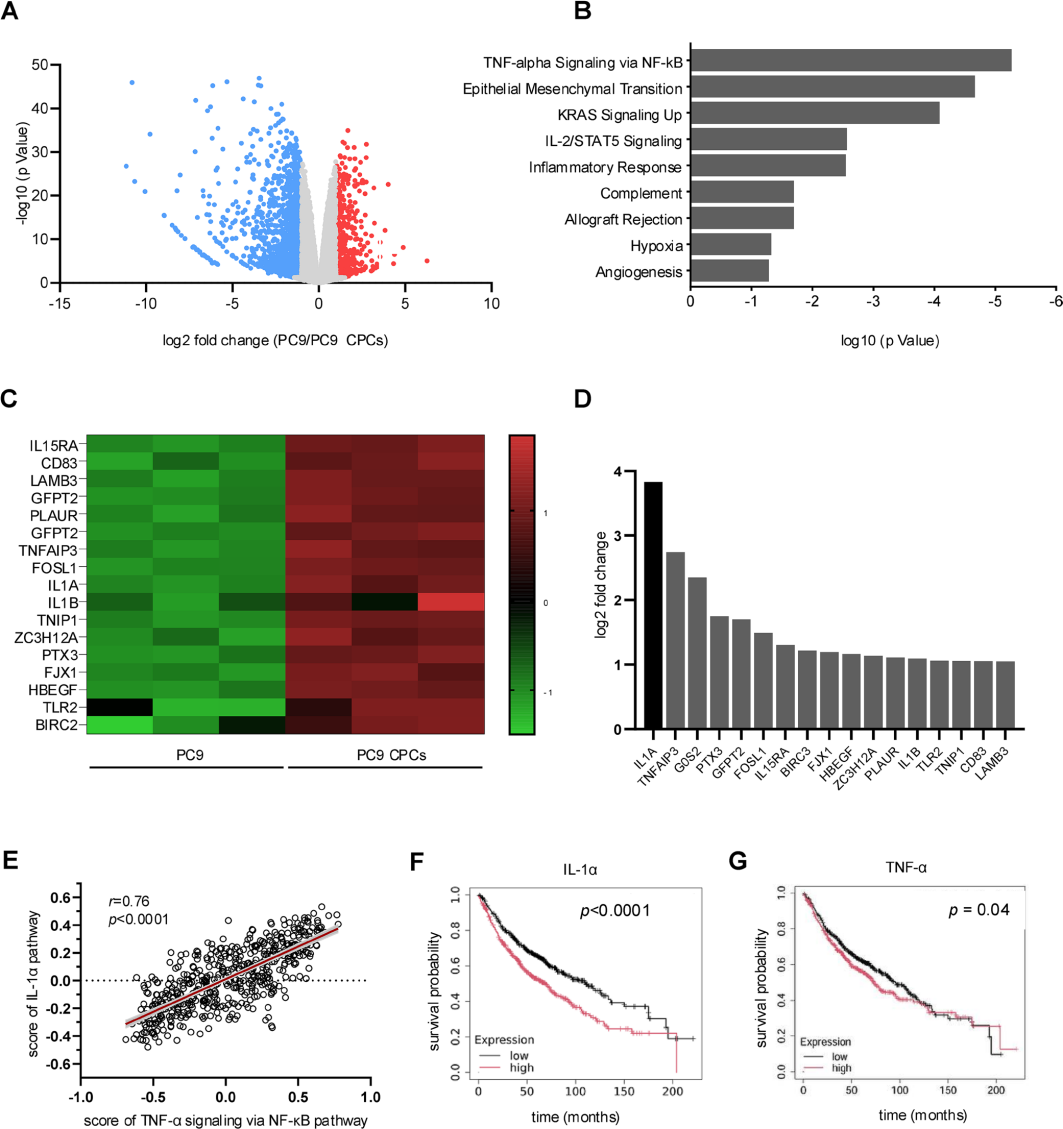
TNF‐α signaling via NF‐ĸB gene signature was linked to osimertinib resistance. (A) Volcano plot of DEGs between PC9‐CPCs and PC9 parental cells. Using cutoff points of log FC > 1 and *p* value ≤ 0.05, 477 upregulated genes in PC9‐CPCs were identified. (B) Enrichment analysis using Enrichr showed that TNF‐α signaling via NF‐ĸB was the most dominant pathway in PC9‐CPCs. (C) Heatmap of the 17 genes identified as likely playing pivotal roles in NF‐κB‐mediated activation of the TNF‐α signaling in PC9 CPCs. (D) The *IL1A* gene had the most significant log FC among all the genes included in the TNF pathway. (E) GSVA was performed to calculate the IL‐1α pathway and TNF pathway scores based on RNA‐seq data from 515 LUAD samples in TCGA. Correlation analysis showed a high correlation between the IL‐1α pathway score and the TNF pathway score. (F‐G) Kaplan–Meier survival analysis of patients with LUAD in the TCGA dataset, stratified by (F) *IL1A* and (G) *TNF* expression levels. CPCs, cycling cancer persister cells; DEGs, differentially expressed genes; GSVA, gene set variation analysis; TNF‐α, tumor necrosis factor alpha; NF‐ĸB, nuclear factor kappa‐light‐chain‐enhancer of activated B cells; *IL1A*, interleukin‐1 alpha; FC, fold change; LUAD, lung adenocarcinoma; TCGA, The Cancer Genome Atlas.

To explore the biological mechanisms that confer resistance against osimertinib treatment, we compared the transcriptomes of PC9‐CPCs and PC9 parental cells by RNA sequencing (RNA‐seq) to obtain the global gene expression profile. A total of 477 genes were found to be upregulated in PC9‐CPCs compared with the control parental cells (Figure 2A). To identify functionally enriched pathways, enrichment analysis was performed using the upregulated genes. The analysis revealed that the TNF‐α pathway via NF‐κB was the most significantly upregulated in osimertinib‐resistant PC9‐CPCs (Figure 2B). Further pathway analysis showed that the epithelial–mesenchymal transition, KRAS signaling, IL‐2‐STAT5 signaling, and inflammatory response pathways were also enriched in PC9‐CPCs.

Subsequent analysis identified 17 genes that likely play pivotal roles in NF‐κB‐mediated activation of the TNF‐α pathway (Figure 2C). Among them, *IL‐1α* was the most significantly upregulated gene (Figure 2D). We hypothesized that tumor‐derived and immune cell‐derived IL‐1α within the tumor microenvironment could cooperatively amplify TNF‐α signaling via NF‐κB. To investigate the relationship between IL‐1α expression and TNF‐α pathway activation, GSVA was applied to publicly available RNA‐seq datasets from 515 lung adenocarcinoma samples in The Cancer Genome Atlas (TCGA) and 44 lung adenocarcinoma cell lines in the cancer cell line encyclopedia. A significant positive correlation was seen between IL‐1α expression and TNF‐α–NF‐κB pathway activity in both cohorts (Figure 2E,S1). To assess the prognostic impact of IL‐1α and TNF‐α in lung adenocarcinoma, we analyzed TCGA survival data using Kaplan–Meier analysis. Elevated *IL‐1α* and *TNF‐α* expression were each significantly associated with reduced OS compared with low‐expression groups (Figure 2F,G).

The findings collectively suggested that activation of the TNF‐α–NF‐κB signaling axis, potentially driven by IL‐1α, contributed to the acquisition of osimertinib resistance in *EGFR*‐mutant lung cancer cells.

### 3.3 Upregulation of IL‐1α Was Associated With NF‐κB p65 Activation, TNF‐α Signaling, and Osimertinib Resistance

**FIGURE 3:**
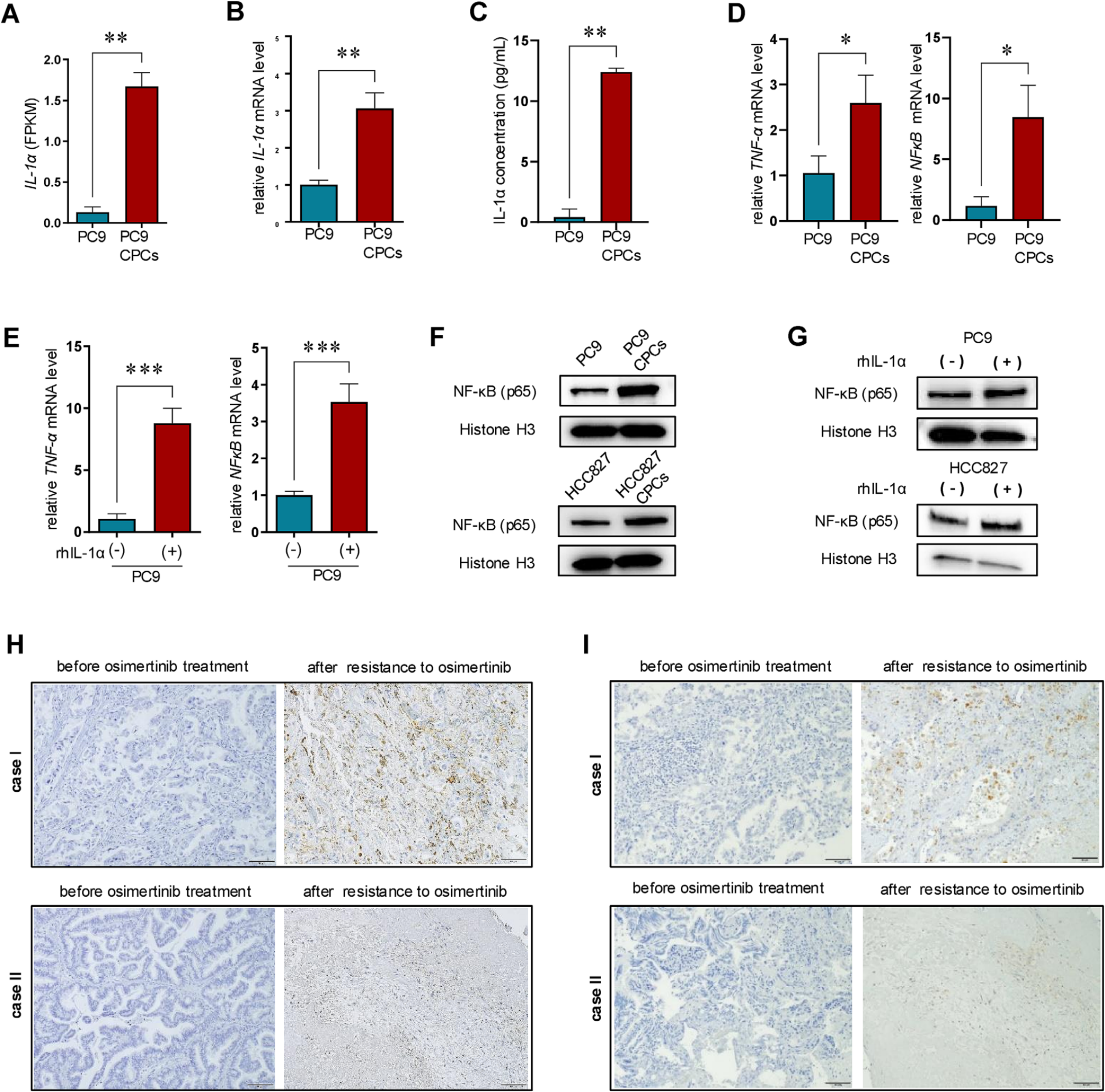
Involvement of IL‐1α, TNF, and NF‐ĸB in osimertinib resistance. (A) RNA‐seq data showing that *IL‐1α* expression was significantly increased in PC9‐CPCs compared to parental cells. (B and C) Validation of *IL‐1α* upregulation in PC9‐CPCs via (B) qPCR and (C) ELISA assay of culture supernatants. (D) qPCR results showing increased *TNF* (left) and *NF‐ĸB* (right) expression in PC9‐CPCs. (E) qPCR results showing that treatment with recombinant IL‐1α for 24 h increased the mRNA expression of *TNF* (left) and *NF‐ĸB* (right) in PC9 parental cells. (F) Western blot analysis showing significantly higher nuclear NF‐ĸB p65 levels in both PC9‐CPCs and HCC827‐CPCs compared to parental cells. (G) Western blot analysis of nuclear NF‐ĸB p65 levels in PC9 and HCC827 parental cells after treatment with recombinant IL‐1α. (H and I) Representative images of immunohistochemical staining showing upregulation of (H) NF‐κB p65 and (I) IL‐1α in tumor tissues after the development of osimertinib resistance. For each panel, the left image shows the specimen before osimertinib treatment, and the right image shows the specimen after the development of osimertinib resistance. Data are presented as mean ± SD and are representative of three independent experiments. *p < 0.05, **p < 0.01, ***p < 0.001, ****p < 0.0001. ELISA, enzyme‐linked immunosorbent assay; IL‐1α, interleukin‐1 alpha; TNF‐α, tumor necrosis factor alpha; NF‐ĸB, nuclear factor kappa‐light‐chain‐enhancer of activated B cells; CPCs, cycling cancer persister cells; qPCR, quantitative polymerase chain reaction; SD, standard deviation.

IL‐1α is a potent activator of the IL1 receptor type 1 (IL1R1) [18]. Its interaction with IL1R leads to the stabilization and nuclear translocation of NF‐κB p65, subsequently inducing the transcription of several pro‐inflammatory cytokines, including TNF‐α. RNA‐seq results demonstrated that IL‐1α was overexpressed in osimertinib‐resistant PC9‐CPCs (Figure 3A). To validate these findings, we performed qPCR using PC9‐CPCs and another resistant line derived from *EGFR*‐mutated lung adenocarcinoma cell line HCC827 (Figures 3B and S2A). Furthermore, ELISA results revealed a significant increase in the level of IL‐1α in both PC9‐CPCs and HCC827‐CPCs (Figures 3C and S2B). Consistently, qPCR analysis showed that TNF‐α and NF‐κB were upregulated in osimertinib‐resistant CPCs (Figure 3D), a finding also observed in HCC827‐CPCs (Figure S2C). To assess whether IL‐1α activates TNF‐α signaling, we treated PC9 and HCC827 parental cells with exogenous IL‐1α for 24 h. As anticipated, the expression of TNF‐α and NF‐κB significantly increased following IL‐1α treatment (Figures 3E and S2D). To verify NF‐κB activity in osimertinib‐resistant CPCs, we performed western blot analysis of NF‐κB p65 in the nuclear fraction of osimertinib‐sensitive and osimertinib‐resistant cells. The nuclear expression of NF‐κB p65 was remarkably higher in resistant cells than in parental cells (Figure 3F). Similarly, treatment with recombinant IL‐1α significantly increased nuclear accumulation of NF‐κB p65 (Figure 3G). To further explore the clinical relevance of these findings, immunohistochemical analysis was performed on paired pre‐treatment and post‐resistance tumor specimens from four patients. Upregulation of NF‐κB p65 and IL‐1α was observed in a subset of cases (2 of 4), suggesting that this inflammatory axis may contribute to osimertinib resistance in some patients (Figure 3H,I). While the limited cohort precludes definitive conclusions regarding the prevalence of this mechanism, the observation is consistent with our in vitro and *in silico* findings. Taken together, the results suggested that IL‐1α possibly acts upstream of TNF‐α signaling through NF‐κB p65 activation in osimertinib‐resistant *EGFR*‐mutant lung cancer cells.

### 3.4 Inhibition of IL‐1α in Osimertinib‐Resistant PC9 Cells Restored Sensitivity to Osimertinib

**FIGURE 4:**
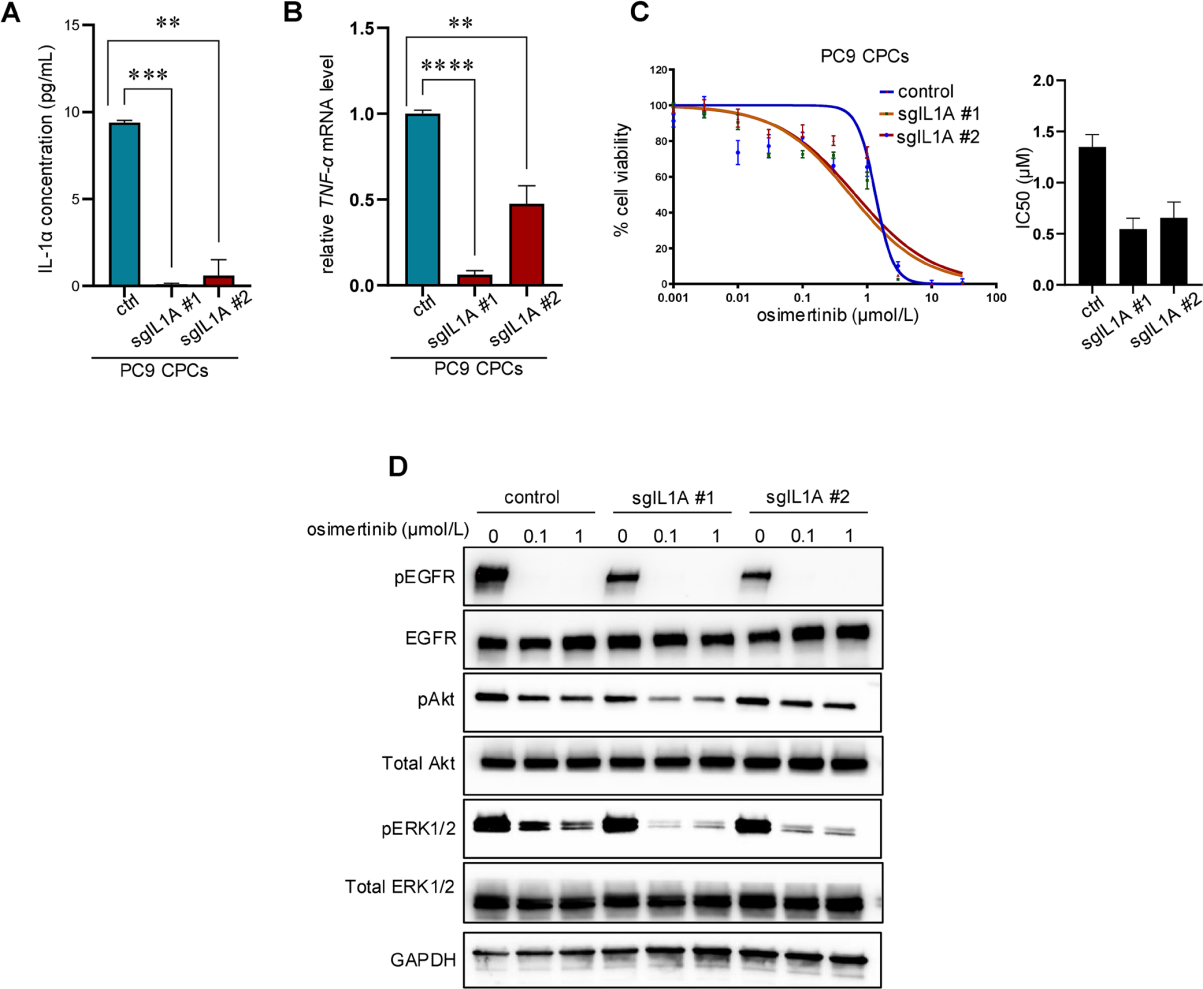
IL‐1α knockout sensitized CPCs to osimertinib. (A) The IL‐1α concentration in the culture supernatant of control and IL‐1α‐knockout (sgIL1A #1 and #2) PC9‐CPCs was measured using ELISA. (B) Relative *TNF* mRNA levels in control and IL‐1α‐knockout (sgIL1A) CPCs, showing significant downregulation of *TNF* following IL‐1α deletion. (C) Cell viability curves and IC_50_ values of osimertinib in control and IL‐1α‐knockout PC9‐CPCs treated for 72 h. (D) Western blot profiling of EGFR–Akt–ERK pathway activity in control and IL‐1α‐knockout PC9‐CPCs treated with increasing doses of osimertinib. Data are presented as mean ± SD and are representative of three independent experiments. **p < 0.01, ***p < 0.001, ****p < 0.0001. IL‐1α, interleukin‐1 alpha; CPCs, cycling cancer persister cells; ELISA, enzyme‐linked immunosorbent assay; TNF‐α, tumor necrosis factor alpha; EGFR–Akt–ERK, epidermal growth factor receptor–protein kinase B–extracellular signal‐regulated kinase; SD, standard deviation.

To determine the role of IL‐1α in the development of osimertinib resistance, the IL‐1α gene in osimertinib‐resistant PC9 and HCC827 cells was knocked out using the CRISPR‐Cas9 system. The efficiency of the knockout was evaluated using ELISA of the culture supernatants (Figures 4A and S3A). We confirmed the downregulation of *TNF‐α* mRNA in IL‐1α‐knockout CPCs of both PC9 and HCC827 cells (Figures 4B and S3B). As expected, the IC_50_ of osimertinib was restored following the IL‐1α knockout (Figure 4C). A similar result was observed in osimertinib‐resistant HCC827 cells (Figure S3C). To further evaluate the effect of IL‐1α deletion on osimertinib‐resistant CPCs, we performed western blot analysis, which showed that osimertinib treatment of IL‐1α‐knockout PC9‐CPCs significantly reduced the expression of phosphorylated ERK and AKT compared with that in control PC9 CPCs (Figure 4D). The results suggested that IL‐1α plays a critical role in mediating osimertinib resistance.

### 3.5 TNF‐α Inhibition Enhanced Osimertinib Sensitivity

**FIGURE 5:**
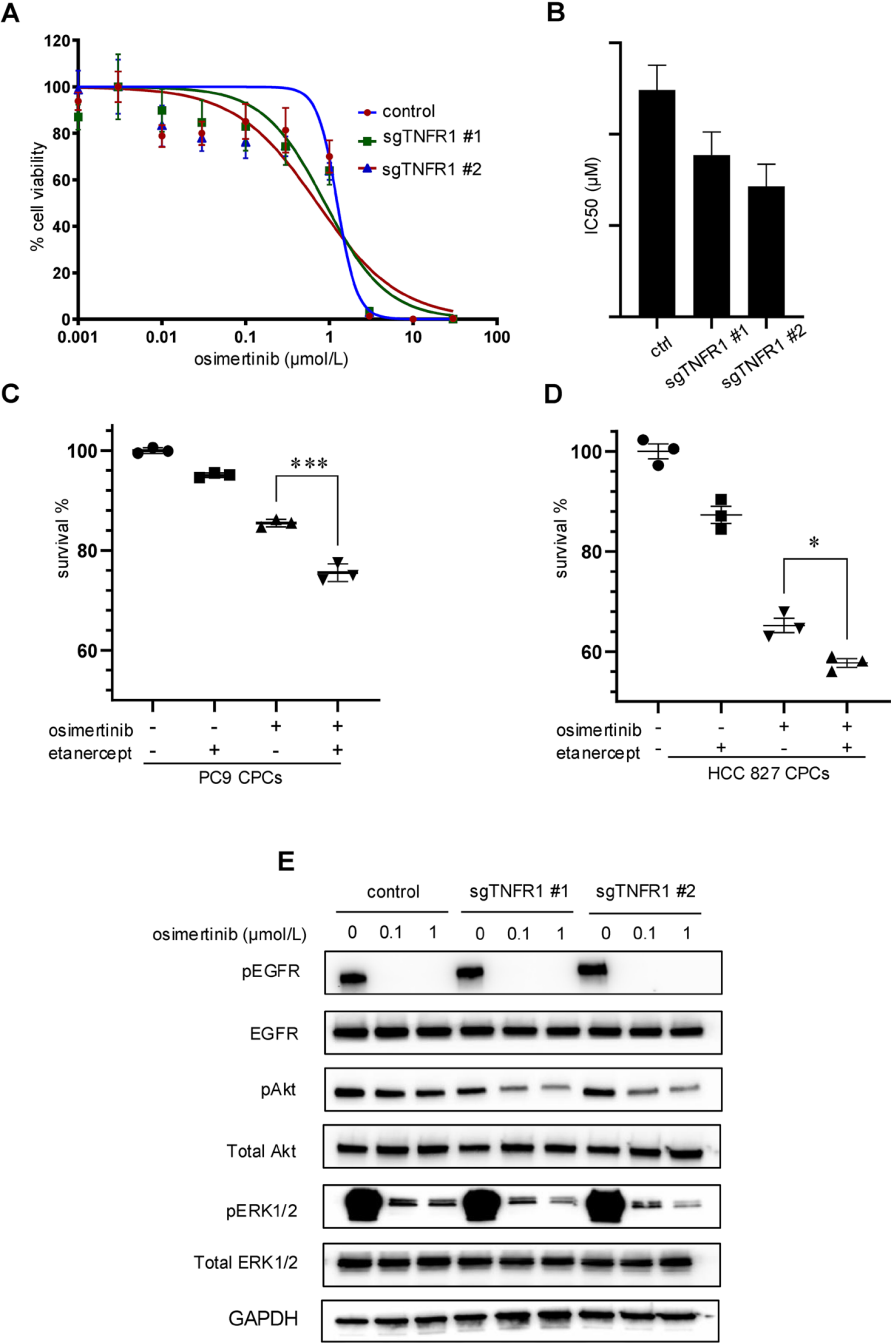
TNF‐α inhibition improved the sensitivity to osimertinib (A and B). (A) Survival curves and (B) IC_50_ values of osimertinib in control and *TNFR1*‐knockout PC9‐CPCs. (C‐D) Cell viability of (C) PC9‐CPCs and (D) HCC827‐CPCs treated with osimertinib in the absence or presence of etanercept (100 μg/mL). (E) Western blot analysis of EGFR–Akt–ERK signaling in control and *TNFR1*‐knockout PC9‐CPCs treated with indicated concentrations of osimertinib. Data are presented as mean ± SD and are representative of three independent experiments. *p < 0.05, ***p < 0.001. TNF‐α, tumor necrosis factor alpha; CPCs, cycling cancer persister cells; EGFR–Akt–ERK, epidermal growth factor receptor–protein kinase B–extracellular signal‐regulated kinase; SD, standard deviation.

Recent findings suggested that TNF‐α signaling contributes to resistance to the first‐generation EGFR‐TKI, erlotinib, and that inhibition of TNF‐α sensitizes NSCLC cells to erlotinib [19, 20]. To determine whether TNF‐α inhibition increases sensitivity to the third‐generation EGFR‐TKI osimertinib in CPCs, both genetic and pharmacological approaches were used to inhibit the TNF‐α pathway. Specifically, we used etanercept, a TNF‐α inhibitor, and performed targeted deletion of the *TNFR1* gene using the CRISPR‐Cas9 system. Our findings indicated that *TNFR1* knockout enhanced the sensitivity of osimertinib‐resistant PC9‐CPCs to osimertinib (Figure 5A,B). Furthermore, viability assays showed increased sensitivity to osimertinib in osimertinib‐resistant cells following etanercept treatment in PC9‐CPCs (Figure 5C). A similar enhancement in sensitivity was observed in osimertinib‐resistant HCC827‐CPCs (Figure 5D). Western blot analysis was performed to examine the effect of TNF‐α inhibition on PC9 CPCs, and the results demonstrated a remarkable decrease in phosphorylated ERK and AKT levels in *TNFR1* knockout PC9‐CPCs (Figure 5E). Taken together, the results suggested that TNF‐α signaling plays a role in conferring resistance to osimertinib in resistant cells.

### 3.6 Inhibition of TNF‐α Increased Sensitivity to Osimertinib In Vivo

**FIGURE 6:**
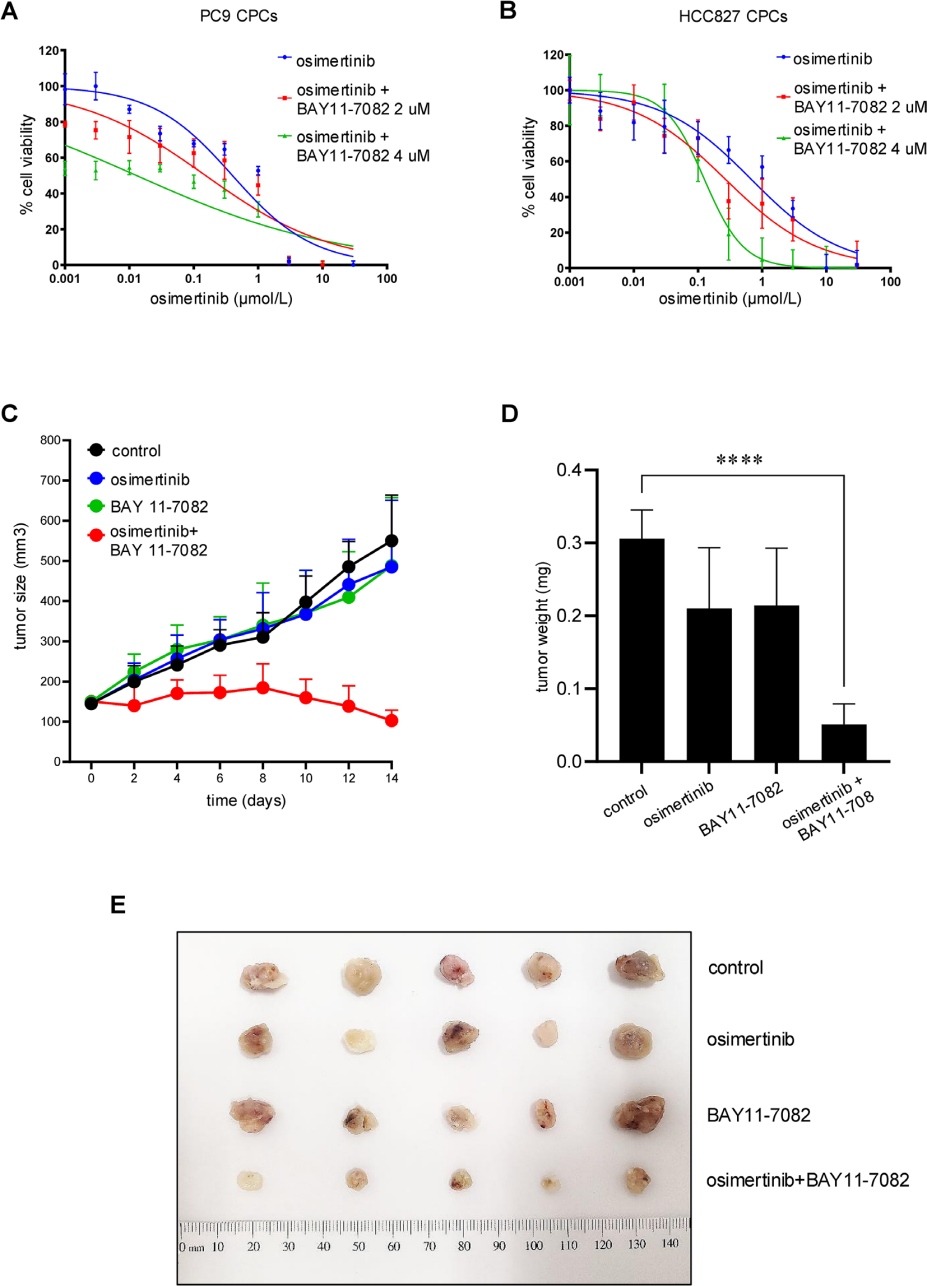
Combined treatment with osimertinib and BAY11‐7082 suppressed tumor growth (A and B). Viability of (A) PC9 CPCs and (B) HCC827 CPCs treated with increasing concentrations of BAY11‐7082 and osimertinib, as measured using CCK‐8 assay. (C) Tumor volume progression in mice bearing PC9‐CPC xenografts treated with vehicle (black), osimertinib alone (blue), BAY11‐7082 alone (green), and combination of osimertinib and BAY11‐7082 (red) (*n* = 5 mice per group). (D) Final tumor weights from each group at the end of the treatment period. (E) Representative images of the excised tumors from each treatment group. Data are presented as mean ± SD. ****p < 0.0001. CPCs, cycling cancer persister cells; CCK‐8, cell counting kit‐8; SD, standard deviation.

To evaluate the biological significance of TNF signaling inhibition in the osimertinib treatment response, in vitro treatment‐response assays were performed in parallel with in vivo experiments, in which PC9‐CPCs were implanted subcutaneously into the flanks of nude mice. Combination treatment with osimertinib and BAY11‐7082, a pharmacological inhibitor of NF‐κB–dependent TNF signaling, demonstrated superior anti‐proliferative efficacy compared with osimertinib monotherapy in both PC9‐CPCs and HCC827‐CPCs. The addition of BAY11‐7082 enhanced growth inhibition in a dose‐dependent manner, indicating that BAY11‐7082 potentiates osimertinib sensitivity and more effectively suppresses cell viability across increasing BAY11‐7082 concentrations (Figure 6A,B). In mice bearing PC9‐CPC xenografts, the combination of BAY11‐7082 and osimertinib resulted in tumors that exhibited significantly slower growth than those in control groups or those treated with either agent alone (Figure 6C–E). These findings indicated that combining BAY11‐7082 with osimertinib could overcome resistance to osimertinib in this cell line. Furthermore, no discernible toxic effects, such as skin rash, infections, or changes in body weight or general condition, were observed in the mice receiving the combination therapy. The findings suggested that administration of the TNF inhibitor BAY11‐7082 could enhance the efficacy of osimertinib in PC9‐CPC cells, as observed in vivo.

## 4 Discussion

In this study, we identified a mechanistic link between osimertinib resistance and activation of the TNF‐α/NF‐κB signaling pathway in CPCs derived from *EGFR*‐mutant NSCLC lines. Our findings demonstrated that IL‐1α functions as a key upstream regulator of this pathway, driving pro‐inflammatory signaling associated with drug tolerance. Notably, inhibition of IL‐1α or TNF‐α restored sensitivity to osimertinib, revealing a previously unrecognized therapeutic vulnerability that could be exploited to overcome resistance in *EGFR*‐mutant lung cancer.

While most cells in TKI‐sensitive NSCLC lines undergo apoptosis when exposed to lethal concentrations of EGFR‐TKIs, a small fraction can survive in a drug‐tolerant state. Sharma and colleagues first described these DTPs in PC9 cells, and further identified proliferating subpopulations named “drug‐tolerant expanding persisters,” which likely correspond to the recently defined CPCs [8, 9]. Our CPC model derived from PC9 and HCC827 cells could provide direct experimental evidence that such persisters can adapt to sustained osimertinib treatment through the activation of inflammatory signaling. Unlike cases of “early resistance,” which arise from preexisting mutations, such as *EGFR* C797S or MET amplification, our whole‐exome sequencing detected no such mutation, suggesting that CPCs represent a non‐genetic, adaptive mechanism underlying the late resistance. Although some studies have not observed CPC‐like behavior, possibly due to limited observation periods or culture conditions [21], our findings supported the existence of a rare subpopulation capable of maintaining proliferative capacity under continuous drug pressure. Further characterization of CPCs is essential to elucidate how they evade TKI therapy and to identify strategies for their complete eradication.

Our transcriptomic analysis revealed prominent activation of the TNF‐α/NF‐κB signaling pathway in osimertinib‐resistant CPCs, with IL‐1α acting as a central upstream mediator. IL‐1α upregulation promoted NF‐κB activation and the subsequent induction of pro‐inflammatory cytokines, including TNF‐α, thereby sustaining resistance‐associated signaling. The results are consistent with previous studies linking TNF‐α to tumor progression and EGFR‐TKI resistance in NSCLC [20], and further indicated that the pathway contributes directly to osimertinib resistance. Although the role of IL‐1α in *EGFR*‐mutant NSCLC has not been well characterized, accumulating evidence suggests that IL‐1α can promote drug resistance, making it a potential therapeutic target in multiple malignancies [19, 22, 23, 24, 25]. Mechanistically, we observed persistent activation of ERK/MAPK and AKT pathways in CPCs despite osimertinib treatment, both of which were attenuated by IL‐1α or TNF‐α inhibition. Furthermore, IL‐1α stimulation increased TNF‐α expression, whereas IL‐1α knockout suppressed it, supporting a model in which IL‐1α functions upstream of TNF‐α/NF‐κB signaling to maintain osimertinib resistance. It is important to note that the CPC phenotype emerges only after sustained osimertinib exposure (approximately 90 days) and reflects cumulative transcriptional and likely epigenetic adaptations. While IL‐1α stimulation of parental cells acutely activates downstream TNF‐α/NF‐κB signaling, short‐term cytokine exposure alone would not be expected to recapitulate the full drug‐tolerant state of CPCs. Rather, our data support a model in which chronic, autocrine IL‐1α signaling progressively shapes a resistance‐permissive transcriptional program that can be reversed by genetic or pharmacological disruption of the axis. Consistent with this concept, a recent study reported that combining a third‐generation EGFR‐TKI with TNF‐α blockade exerted synergistic anti‐tumor effects in *EGFR*‐mutant NSCLC [26]. Our findings build upon and extend this prior work in three important respects. First, we identify IL‐1α as the upstream driver of TNF‐α/NF‐κB activation in osimertinib‐resistant CPCs, providing a more proximal and potentially more druggable target. Second, we demonstrate that this inflammatory circuit operates through an autocrine, tumor cell–intrinsic mechanism, distinct from paracrine signaling typically attributed to stromal or immune‐derived TNF‐α. Third, we provide evidence that pharmacological inhibition of downstream NF‐κB also reverses resistance in vivo, supporting multiple actionable nodes along the IL‐1α–TNF‐α–NF‐κB axis.

Inflammatory cytokines are known contributors to tumor progression and resistance to EGFR‐TKIs, largely through paracrine signaling from immune and stromal cells within the tumor microenvironment [27]. They promote survival signaling, epithelial–mesenchymal transition, and stemness, thereby reducing the efficacy of EGFR‐targeted therapy [28]. Our current study highlighted an additional mechanism beyond paracrine signaling, via autocrine cytokines produced directly by tumor cells. We found that IL‐1α and TNF‐α were upregulated in osimertinib‐resistant cell lines and were associated with resistant phenotypes. The cytokines sustained oncogenic signaling and enabled cellular adaptations that diminished the inhibitory effects of EGFR blockade. The observation supported a paradigm in which tumor cells actively remodel their own microenvironment through cytokine secretion to evade targeted therapy. Targeting such autocrine cytokine loops could, therefore, represent a promising strategy to overcome or delay resistance to osimertinib in *EGFR*‐mutant NSCLC.

Although IL‐1α, TNF‐α, and NF‐κB all represent viable therapeutic targets within the axis we describe, several considerations favor IL‐1α blockade as the most clinically promising candidate. First, in terms of druggability and clinical availability, anakinra (a recombinant IL‐1 receptor antagonist) and canakinumab (an anti–IL‐1β monoclonal antibody, with related agents under development against IL‐1α) are already approved for inflammatory conditions and have well‐characterized safety profiles in patients, including in oncology trials such as CANTOS [29]. Second, owing to its upstream position, targeting IL‐1α offers the potential to simultaneously block multiple downstream effectors—including TNF‐α, NF‐κB, and other inflammatory cytokines—potentially providing a broader inhibitory effect than blocking individual downstream nodes. Third, with regard to safety, while TNF‐α inhibitors such as infliximab and etanercept are clinically established, they carry well‐known risks of serious infection and reactivation of latent infections in cancer patients undergoing systemic therapy. NF‐κB inhibitors, although mechanistically attractive, have to date faced challenges in clinical translation due to on‐target toxicity arising from the pleiotropic roles of NF‐κB in homeostasis. Taken together, we propose that IL‐1α–directed agents merit prioritization in future clinical investigations as combination partners with osimertinib in *EGFR*‐mutant NSCLC.

The more pronounced combination effect of NF‐κB inhibition with osimertinib observed in vivo compared with in vitro likely reflects several factors. First, with respect to duration of exposure, in vivo tumors received continuous treatment for 2 weeks, allowing the cumulative impact of NF‐κB inhibition on tumor cell survival, proliferation, and adaptive transcriptional responses to manifest, whereas in vitro CCK‐8 assays assessed viability after only 72 h. Second, regarding microenvironmental contributions, in vivo tumors are exposed to additional sources of TNF‐α and other NF‐κB–activating cytokines derived from stromal and innate immune cells (which remain functional even in nude mice), so NF‐κB inhibition disrupts both autocrine and paracrine pro‐survival signals; in vitro monocultures lack this paracrine input, limiting the apparent benefit of NF‐κB blockade. Third, additional NF‐κB–dependent processes operative in vivo—including angiogenesis, extracellular matrix remodeling, and anti‐apoptotic gene expression—are absent in two‐dimensional culture. Together, these factors plausibly account for the enhanced combination effect observed in the xenograft model and underscore the clinical relevance of the in vivo findings.

The current study has some limitations. First, although we demonstrated that combining osimertinib with an NF‐κB inhibitor suppressed tumor growth in vivo, we did not directly evaluate IL‐1α or TNF‐α blockade in animal models. The study was primarily designed to elucidate the mechanistic role of IL‐1α–mediated TNF‐α/NF‐κB signaling in CPCs and to provide proof‐of‐concept evidence for therapeutic targeting. Future studies should aim to determine whether direct inhibition of IL‐1α or TNF‐α can reproduce the effects in xenograft or patient‐derived models, which would be essential to translate our findings into potential clinical applications. Second, the number of patient tumor specimens analyzed was limited (*n* = 4 paired samples), and only a subset showed upregulation of NF‐κB p65 and IL‐1α. These immunohistochemical observations should therefore be regarded as hypothesis‐generating rather than conclusive evidence of clinical prevalence. Validation in larger, prospectively collected cohorts—ideally incorporating quantitative protein measurements and correlation with clinical outcomes—will be necessary to determine the frequency and clinical impact of IL‐1α–driven TNF‐α/NF‐κB signaling in osimertinib‐resistant tumors. Third, we did not evaluate the tumor microenvironment comprehensively. The TNF‐α/NF‐κB axis may also be activated by cytokines derived from stromal or immune cells; thus, further studies incorporating both tumor‐intrinsic and microenvironmental factors would be necessary to fully delineate the contribution of IL‐1α–mediated inflammatory signaling to resistance mechanisms. Fourth, our study compared CPCs with parental cells but did not include direct comparisons with intrinsic or genetically acquired resistance models. Therefore, whether the IL‐1α–TNF‐α/NF‐κB axis represents a unique vulnerability of the CPC state, or a broader feature shared with other stress‐induced resistance phenotypes, remains to be determined. Future studies incorporating intrinsic resistance models and acquired resistance models harboring known genetic alterations (e.g., T790M, C797S, MET amplification) will be essential to delineate the specificity of this axis. Fifth, during the generation of *IL1A*‐ and *TNFR1*‐knockout and control CPC lines, cells were transiently cultured without osimertinib during lentiviral transduction and puromycin selection. Because IL‐1α expression in CPCs is sustained by continuous osimertinib pressure, this brief drug‐free interval may have attenuated baseline IL‐1α expression in the control group, potentially narrowing the apparent difference between control and knockout cells and contributing to the partial suppression of pERK observed even in control CPCs upon osimertinib re‐challenge. Importantly, however, both groups underwent identical handling, so the relative comparison remains internally valid; the observed restoration of osimertinib sensitivity in IL‐1α–knockout CPCs and the marked suppression of downstream signaling demonstrate the functional requirement of IL‐1α for the resistant phenotype. Sixth, an important question raised by our findings is whether genetic ablation of *IL1A* in parental *EGFR*‐mutant cells would prevent or delay the emergence of CPCs under continuous osimertinib pressure. Because our data demonstrate that IL‐1α is functionally required to sustain the CPC state, we predict that *IL1A* knockout in parental cells would impair CPC development. Direct experimental verification of this prediction, including time‐course quantification of persister populations and characterization of any escape mechanisms, represents an important next step that may inform whether IL‐1α blockade should be deployed prophylactically alongside osimertinib initiation, rather than only after resistance emergence. Despite the limitations, our findings provided important mechanistic insights into osimertinib resistance and identified a potential therapeutic vulnerability that warrants further investigation.

In conclusion, we demonstrated that IL‐1α–mediated activation of the TNF‐α/NF‐κB pathway defines a non‐genetic mechanism of resistance to osimertinib in *EGFR*‐mutant NSCLC. The pathway sustains CPCs under continuous drug exposure, enabling tumor survival despite EGFR inhibition. Targeting IL‐1α/TNF‐α–driven NF‐κB signaling could provide a promising therapeutic strategy to overcome resistance to osimertinib and improve clinical outcomes in *EGFR*‐mutant NSCLC.

## Author Contributions

**Wira Winardi:** methodology, investigation, validation, formal analysis, visualization, conceptualization, writing – original draft, writing – review and editing. **Fumiyuki Takahashi:** conceptualization, supervision. **Daisuke Hayakawa:** investigation. **Takuo Hayashi:** investigation, supervision. **Aditya Wirawan:** investigation. **Ikuko Nakamura:** investigation. **Yoichiro Mitsuishi:** conceptualization, methodology, data curation, supervision, formal analysis, investigation, validation, funding acquisition, visualization, project administration, resources, writing – review and editing, writing – original draft, software. **Hironari Matsuda:** investigation, resources. **Moulid Hidayat:** investigation. **Adityo Wibowo:** investigation. **Koichiro Kanamori:** investigation. **Shinji Kohsaka:** investigation, supervision, software. **Ken Tajima:** conceptualization, supervision, funding acquisition, writing – original draft. **Takehito Shukuya:** supervision, writing – original draft. **Kazuhisa Takahashi:** supervision, writing – original draft, writing – review and editing, funding acquisition.

## Funding

This work was supported by Japan Society for the Promotion of Science (JSPS) (21K08187, 23K07636, and 23H02774).

## Disclosure

The authors have nothing to report.

## Ethics Statement

Approval of the research protocol by an Institutional Reviewer Board: Human tissue samples were obtained with approval from the Institutional Review Board of Juntendo University Hospital (Approval No. E24‐0373‐H01). Animal Studies: All animal studies were conducted in accordance with protocols approved by the Animal Care and Use Committee of the Juntendo University School of Medicine (Approval No. 2023274).

## Conflicts of Interest

The authors declare no conflicts of interest.

## Consent

Written informed consent for all genetic and cell biological analyses was obtained from all patients.

## Supporting information


**Figure S1:** Related to Figure 2. GSVA was performed to calculate the IL‐1α pathway and TNF pathway scores based on RNA‐seq data from 44 LUAD cell lines from CCLE. Correlation analysis showed a high correlation between the IL‐1α pathway score and the TNF pathway score.

**Figure S2:** Related to Figure 3. (A) qPCR analysis showed increased *IL‐1α* mRNA expression in HCC827‐CPCs compared with parental HCC827 cells. (B) ELISA of culture supernatants showed increased IL‐1α secretion in HCC827‐CPCs. (C) qPCR results showing increased *TNF* and *NF‐κB* expression in HCC827‐CPCs. (D) qPCR results showing that treatment with recombinant IL‐1α for 24 h increased the mRNA expression of *TNF* (left) and *NF‐κB* (right) in HCC827 parental cells.

**Figure S3:** Related to Figure 4. (A) The IL‐1α concentration in the culture supernatant of control and IL‐1α‐knockout (sgIL1A #1 and #2) HCC827‐CPCs was measured using ELISA. (B) Relative *TNF* mRNA levels in control and IL‐1α‐knockout (sgIL1A) CPCs, showing significant downregulation of *TNF* following IL‐1α deletion. (C) Cell viability curves and IC_50_ values of osimertinib in control and IL‐1α‐knockout HCC827 CPCs.

## Data Availability

The data that support the findings of this study are available from the corresponding author upon reasonable request. Publicly available gene expression and survival datasets analyzed in this study can be accessed through the Kaplan–Meier Plotter (https://kmplot.com).
