# Supplementary figures and images for: Interleukin‐1α‐Mediated Activation of TNF‐α/NF‐κB Signaling Confers Resistance to Osimertinib in EGFR ‐Mutant Non‐Small‐Cell Lung Cancer
Source: Thorac Cancer. 2026 Jul 17;17(14):e70360. doi: 10.1111/1759-7714.70360 (PMC13379369; doi:10.1111/1759-7714.70360)

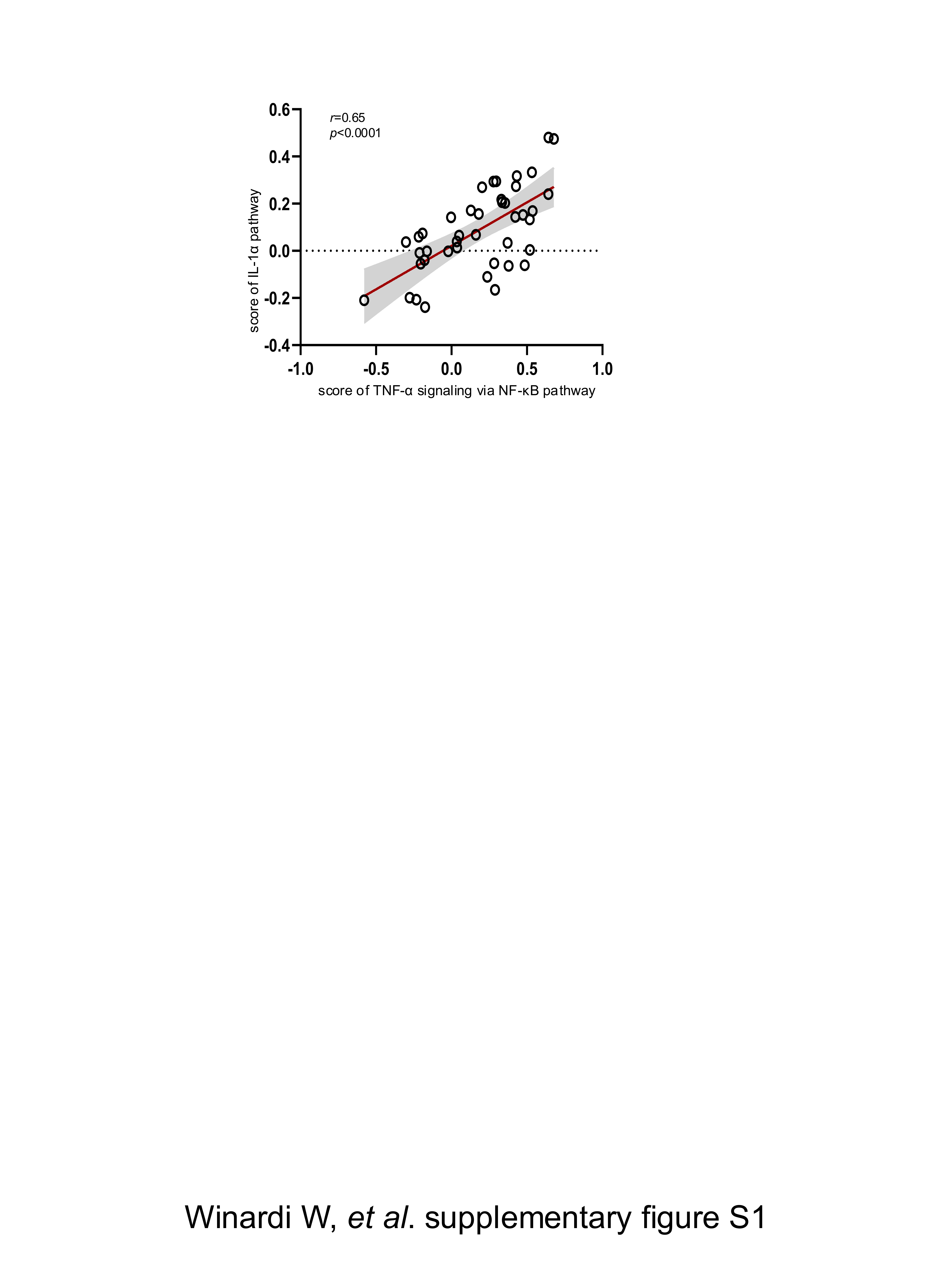

Supplement: Supplementary file 1 — Figure S1: Related to Figure 2. GSVA was performed to calculate the IL‐1α pathway and TNF pathway scores based on RNA‐seq data from 44 LUAD cell lines from CCLE. Correlation analysis showed a high correlation between the IL‐1α pathway score and the TNF pathway score. [file TCA-17-e70360-s002.tiff]

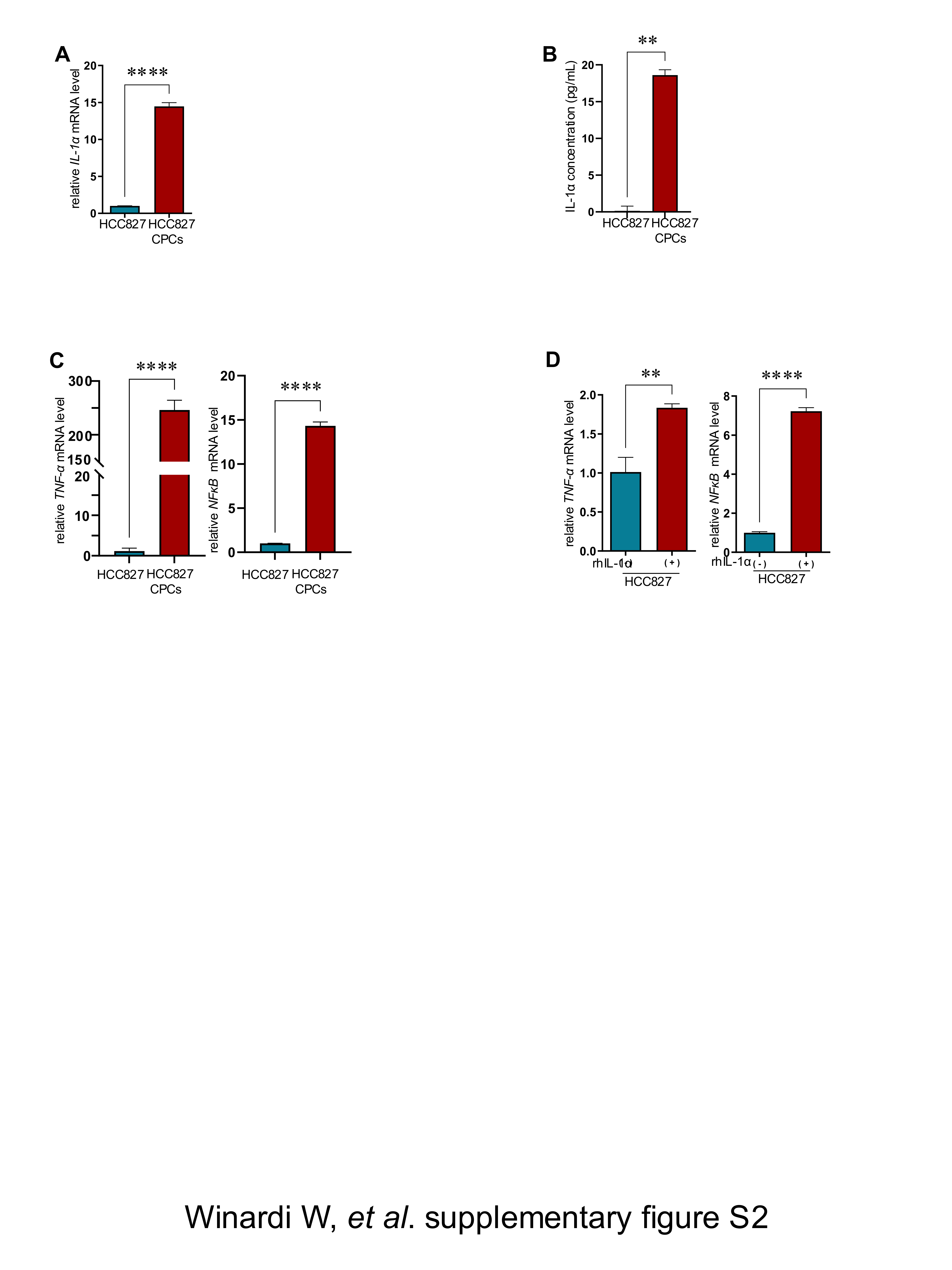

Supplement: Supplementary file 2 — Figure S2: Related to Figure 3. (A) qPCR analysis showed increased IL‐1α mRNA expression in HCC827‐CPCs compared with parental HCC827 cells. (B) ELISA of culture supernatants showed increased IL‐1α secretion in HCC827‐CPCs. (C) qPCR results showing increased TNF and NF‐κB expression in HCC827‐CPCs. (D) qPCR results showing that treatment with recombinant IL‐1α for 24 h increased the mRNA expression of TNF (left) and NF‐κB (right) in HCC827 parental cells. [file TCA-17-e70360-s003.tiff]

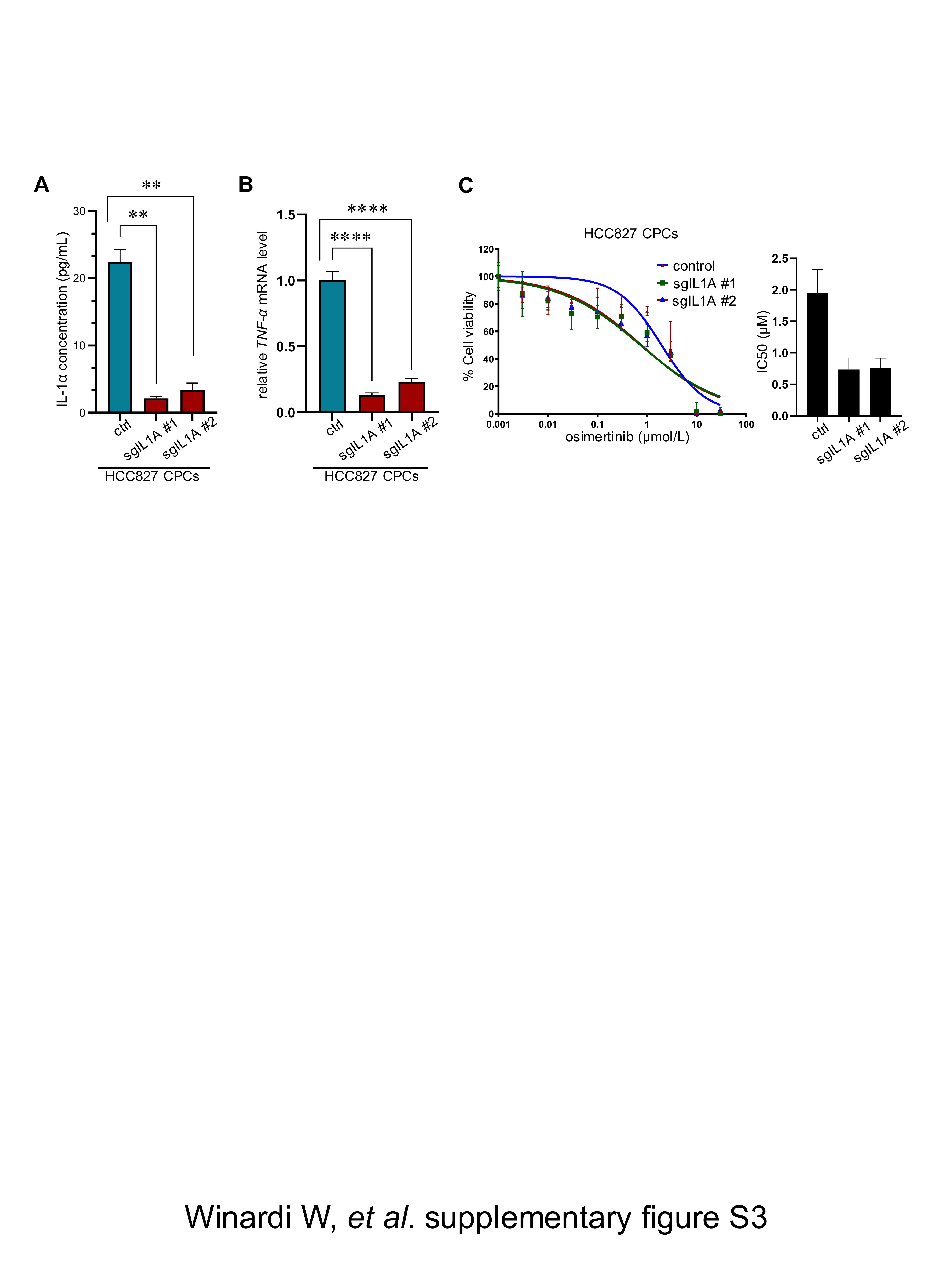

Supplement: Supplementary file 3 — Figure S3: Related to Figure 4. (A) The IL‐1α concentration in the culture supernatant of control and IL‐1α‐knockout (sgIL1A #1 and #2) HCC827‐CPCs was measured using ELISA. (B) Relative TNF mRNA levels in control and IL‐1α‐knockout (sgIL1A) CPCs, showing significant downregulation of TNF following IL‐1α deletion. (C) Cell viability curves and IC50 values of osimertinib in control and IL‐1α‐knockout HCC827 CPCs. [file TCA-17-e70360-s001.tiff]
